# Ex vivo biomechanical evaluation of a bone-screw-fastener for tibial plateau leveling osteotomy

**DOI:** 10.3389/fvets.2023.1207563

**Published:** 2023-06-29

**Authors:** William S. Kettleman, Michael H. Jaffe, Robert W. Wills, Sara J. Dietz, Steve H. Elder

**Affiliations:** ^1^Department of Clinical Sciences, College of Veterinary Medicine, Mississippi State University, Starkville, MS, United States; ^2^Department of Comparative Biomedical Sciences, College of Veterinary Medicine, Mississippi State University, Starkville, MS, United States; ^3^Department of Agricultural and Biological Engineering, Bagley College of Engineering, Mississippi State University, Starkville, MS, United States

**Keywords:** bone-screw-fastener, cranial cruciate ligament, screw, stifle, TPLO

## Abstract

**Introduction:**

The objective of this study was to investigate the effect of a novel screw type on stiffness and failure characteristics of a tibial plateau leveling osteotomy (TPLO) construct under cyclic loading conditions. The authors hypothesized that bone-screw-fasteners (BSF) would result in superior biomechanical stability compared with locking buttress screws (LBS).

**Materials and Methods:**

Twelve pairs of canine cadaveric pelvic limbs were included in this *ex vivo* biomechanical study. A TPLO was performed using a 3.5mm locking TPLO plate and stabilized using either LBS or BSF. Cyclic loading was performed for 30,000 cycles at 4Hz with a peak-load of 1000N (50N valley). The cyclic test was then continued by stepwise incremental increase of peak-load at a rate of 75N per 500 cycles until failure.

**Results:**

Cycles to failure for LBS (44,260 ± 5,770) and BSF (41,540 ± 7,686) were not significantly different (*p* = 0.36). Maximum force for LBS (3,134 ± 797N) and BSF (2,940 ± 831N) was not significantly different either (*p* = 0.58). Dynamic stiffness for LBS (1,778 ± 932 N/mm) and BSF (1,574 ± 677 N/mm) was not significantly different (*p* = 0.58).

**Discussion:**

Stabilization of the TPLO with BSF provided similar biomechanical stability under cyclic axial loading conditions as the LBS. BSF may be an acceptable alternative to traditional locking screws for TPLO.

## Introduction

Cranial cruciate ligament (CrCL) disease is the most common cause of canine pelvic limb lameness (1). The tibial plateau leveling osteotomy (TPLO) was introduced in 1993 as an extra-articular surgical technique for stabilization of the CrCL-deficient stifle joint (2). Neutralization of cranial tibial thrust by reducing the slope of the tibial plateau eliminates the need for an intact CrCL (2–7). A cylindrical-shaped tibial osteotomy is created to level the tibial plateau; following osteotomy, the tibial plateau is rotated about its mediolateral axis until a final tibial plateau angle (TPA) of approximately 5–7° is reached (2, 7). The newly aligned osteotomy is maintained in reduction using a specialized bone plate and screws (8).

Conventional screws used in the TPLO procedure rely on buttress threads developed several decades ago. Reported disadvantages of buttress screws include stripping, screw loosening, creation of a stress riser, bone microfracture, thermal necrosis, and fixation failure with risk of nonunion or malunion. (9) Recently, the bone-screw-fastener or interlocking thread screw was introduced to human orthopedics (9–12). The fastener was designed to create an interlocking bone-screw interface by utilizing a “female thread” bone cutting technology, similar to a nut and bolt (9). This screw theoretically preserves bone integrity, interlocks with bone, resists multidirectional loading forces, and prevents implant loosening. Bone-screw-fasteners have been successfully used in the management of various fractures in humans via open reduction and internal fixation using standard AO plates (10). The fasteners have been shown to perform similarly to traditional locking screws in geriatric female cadaveric tibias and have reported improved biomechanical performance compared to buttress threaded screws in an *ex vivo* adult canine humeral condylar fracture model (12, 13).

To date, there is only one peer-reviewed publication reporting the use of bone-screw-fasteners in small animal orthopedic surgery (13). Because it is unknown how the new fasteners will perform mechanically in a TPLO construct, the objective of this study was to investigate the effect of screw type on stiffness and failure characteristics of a TPLO construct under cyclic loading conditions. The authors hypothesized that bone-screw-fasteners would result in superior biomechanical stability compared with locking buttress screws.

## Materials and methods

### Specimen collection and preparation

**Figure 1:**
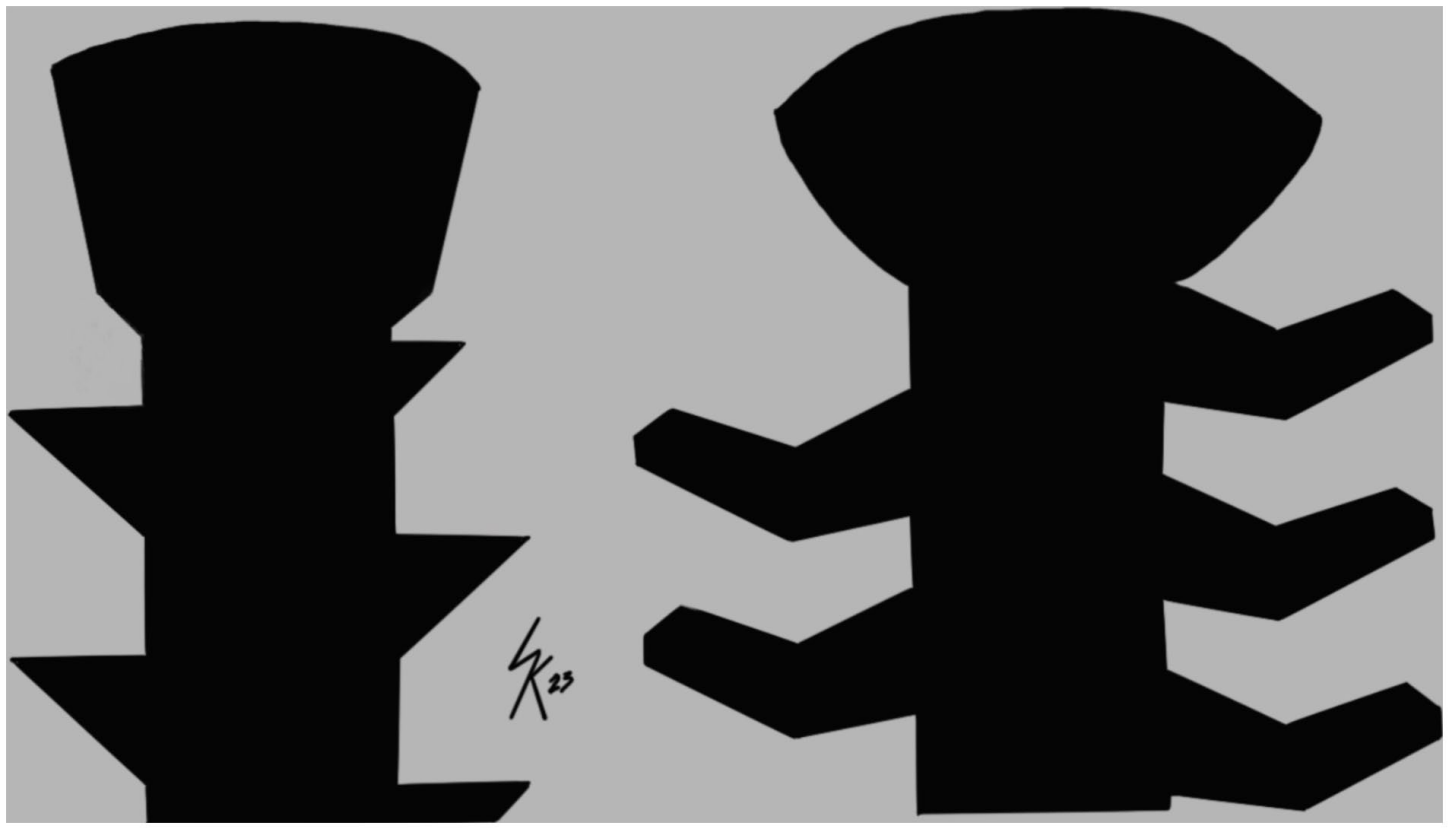
Illustration of thread geometry. Left is a locking buttress screw. Right is a bone-screw-fastener.

Methods of *ex vivo* bone collection and preparation were similar to those previously described (8). Twelve pairs of pelvic limbs were collected from adult dogs euthanized for reasons unrelated to this study. Weight of canine cadavers was not uniformly available. Stifle joints were determined to be anatomically and clinically normal through gross and orthogonal radiographic evaluation. Tibias were dissected en bloc, stripped of soft tissue, and randomly assigned pairwise to two study groups: locking buttress screw (LBS) or bone-screw-fastener (BSF) (Figure 1). Left and right bones were equally distributed within the paired groups. Specimens were stored at −20°C in saline-soaked sponges until testing, at which point they were thawed.

### Measurement of TPA

Preoperative mediolateral radiographs of all specimens were obtained for measurement of TPA as previously described (1). Preoperative orthopedic planning software (vPOP^PRO^; VetSOS Education Ltd.) was used to determine TPA. TPA was measured independently by two experienced surgeons for all specimens, and the results were averaged. TPA of bones in our study ranged from 23° to 30° [LBS: 26.8 ± 2.1° (mean ± SD); BSF: 26.2 ± 2.3°]. A radiographic template for a 24-mm radial osteotomy was centered at the intersection of the tibial plateau and the tibial long axis lines. The distance (D1) from the patellar ligament attachment to the proposed osteotomy was measured along a line perpendicular to the cranial border of the tibia to ensure a tibial tuberosity width of ≥10 mm (14). A commercially available chart (DePuy Synthes Vet) was used to determine the magnitude of rotation of the tibial plateau in millimeters required to achieve a postoperative TPA of 5°.

### TPLO procedure

All TPLO procedures were performed by the same surgeon and assistant surgeon as previously described (1). An oscillating saw (DePuy Synthes Vet) was equipped with a biradial 24-mm TPLO saw blade (DePuy Synthes Vet). Following osteotomy, the tibial plateau segment was rotated the predetermined amount, and temporary reduction was achieved by insertion of a 1.6-mm-diameter Kirschner wire. A pair of pointed bone reduction forceps were applied to provide additional stability and compression of the osteotomy.

A 3.5-mm, 6-hole, locking TPLO plate was used to stabilize the osteotomy. The plate was manufactured from implant grade 316 L stainless steel, with left and right versions anatomically pre-contoured to match the proximal medial tibial surface. Three round, double-threaded holes in the proximal aspect of the plate can accept 3.5-mm screws with or without a locking head and are oriented for optimal bone purchase, and to avoid screw placement within the joint. The distal shaft of the plate has two multipurpose, dynamic compression holes (which allow locking, neutral, or compression screws) and one central hole similar to those in the proximal head of the plate. The underside of the implant has the same configuration as limited contact dynamic compression plates with a scalloped surface designed to reduce contact of the plate with the bone and interference with blood supply. For the purpose of this study, screw holes were labeled proximally to distally 1 through 6.

The LBS group was considered the control group in which 3.5-mm, self-tapping buttress threaded screws were used following AO plating principles. First, the shaft of the plate was attached to the tibial diaphysis with a 3.5-mm cortical screw inserted in the proximal dynamic compression hole (screw hole 4) in neutral position. Next, a 3.5-mm cortical screw was placed in the distal dynamic compression hole (screw hole 6) in the load position, and this screw was not fully tightened. Three 3.5-mm locking-head screws were inserted in the three proximal holes of the plate (screw holes 1–3) and tightened with a star-drive screwdriver shaft and a torque-limiting device (DePuy Synthes Vet) to a maximum of 1.5 Nm. The distal cortical screw (screw hole 6) was tightened until it contacted the plate. Then, the proximal cortical screw (screw hole 4) was slightly loosened, and the distal cortical screw (screw hole 6) was fully tightened by hand to achieve interfragmentary compression of the osteotomy gap. The proximal cortical screw was retightened (screw hole 4). Last, the remaining central hole in the distal shaft of the plate (screw hole 5) was filled with a 3.5-mm locking screw tightened to 1.5 Nm. The Kirshner wire and pointed bone reduction forceps were removed.

In the BSF group, 3.5-mm, self-tapping bone-screw-fasteners were used exclusively. The TPLO plate was applied to the bone by insertion of fasteners in the same order as the control group. Before screw insertion, 2.5-mm pilot holes were drilled in the bone using a specially manufactured drill bit. This drill bit, designed in conjunction with the BSF, theoretically preserves bone volume and improves clearance of bone debris from the drill hole, therefore optimizing the bone-screw interface (10, 13). A torque-limiting device was used for screw insertion. Postoperative radiographs were used to confirm osteotomy position, post-operative TPA, and implant placement. Cranial, caudal, medial, and lateral view photographs of the specimens were acquired (iPhone 12 Pro; Apple Inc.).

### Mechanical testing

**Figure 2:**
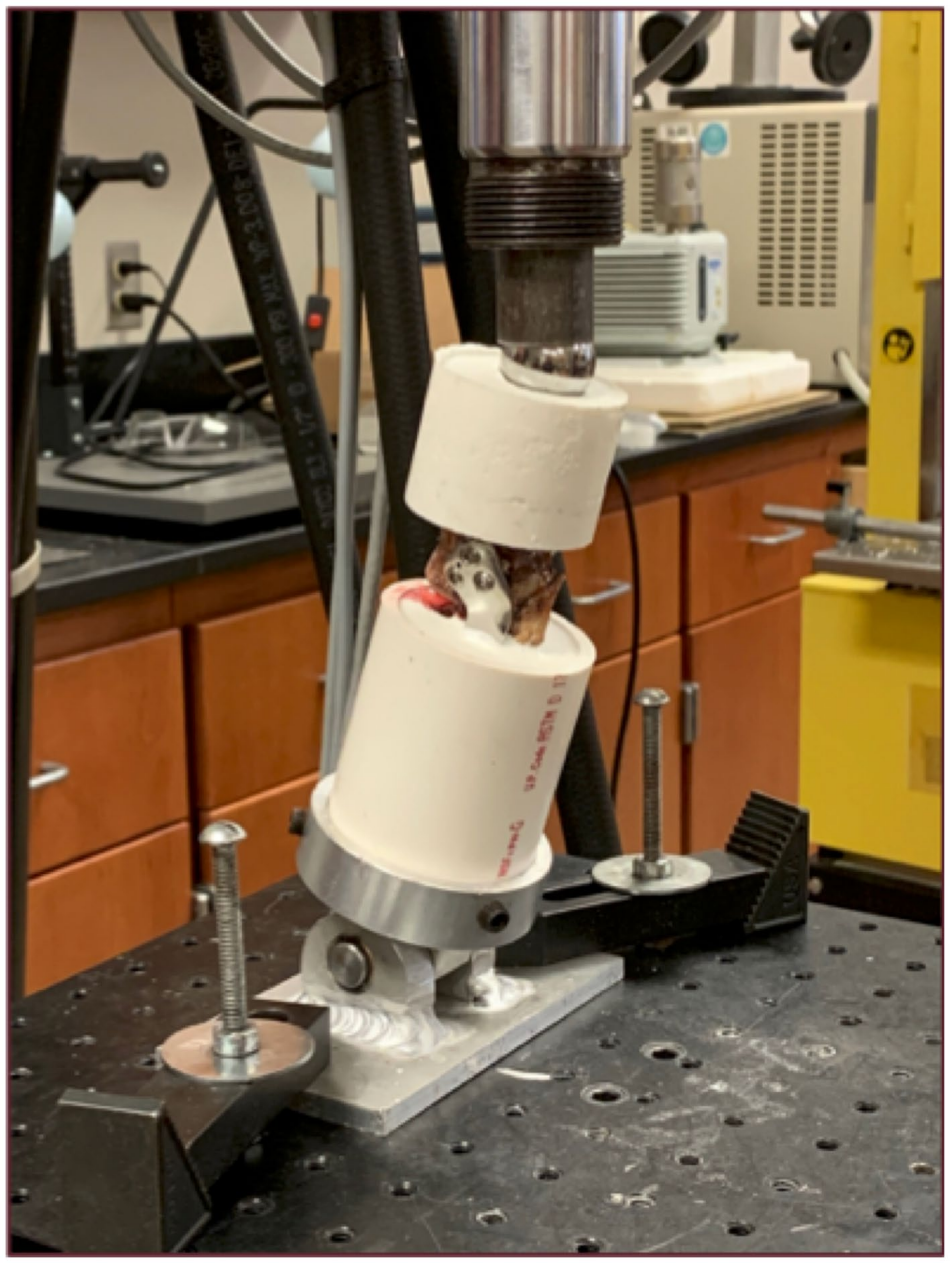
Setup for mechanical testing. Truncated and embedded specimen (left tibia) in testing apparatus at 75° to the horizontal. Load introduction via metal sphere proximally. Metal rod and sleeve bearings distally to prevent rotation.

Following the TPLO procedure, tibias were transected transversely with an oscillating saw 70 mm distal to the distal-most aspect of the osteotomy. The distal 60 mm of bone, plate, and screws were embedded in polyurethane casting resin (Fabri-Cast 50; Specialty Resin & Chemical). The tibial plateau fragment was embedded proximally in polyurethane without encompassing the plate and screws. Each specimen was positioned in a servohydraulic testing machine (MTS Bionix 858 Test System; MTS Systems Corporation) at 75° to the horizontal to simulate physiological orientation and loading at the mid-point of the stance phase at the walk (Figure 2) (7, 8, 15, 16). Load was transferred proximally into the polyurethane via a 25 mm diameter metal sphere placed between the medial and lateral intercondylar tubercles. The distal aspect of the specimen rested on a metal rod and sleeve bearings to prevent rotation. Cyclic loading was performed for 30,000 cycles at 4 Hz in load control with a peak-load of 1,000 N (50 N valley). The cyclic test was then continued by stepwise increasing the peak-load at a rate of 75 N per 500 cycles (0.15 N/cycle) until failure of the construct. Failure was defined as 6 mm crosshead displacement at unloading to 50 N (plastic deformation) (8). Constructs were examined visually post-testing to determine mode of failure. Cycles to failure, maximum force, and dynamic stiffness were determined for all constructs.

### Statistical analysis

The effect of screw type and leg side on cycles to failure, maximum force, and dynamic stiffness was assessed with separate linear mixed models. For each outcome, screw type, leg side, and their interaction were included as fixed effects. If the interaction term was not significant, it was removed, and the model was refit. Similarly, if leg side was subsequently not significant, it was removed and the model refit. Dog identity was included as a random effect. Conditional residuals were visually assessed to determine if the assumptions of normality and homoscedasticity were met for the statistical models. These assumptions were not met by the linear mixed models for dynamic stiffness for cycles with stepwise increasing load and for overall dynamic stiffness. Accordingly, the effect of screw type on these outcomes was assessed using a Wilcoxon signed rank test. Mean and standard deviation were reported for cycles to failure, maximum force, and dynamic stiffness for the first 30,000 cycles; median and interquartile range were reported for dynamic stiffness for cycles with increasing load and overall dynamic stiffness. All statistics were performed using computerized software (SAS Institute Inc.). An alpha level of 0.05 was used to determine statistical significance for each of the statistical methods.

## Results

**Figure 3:**
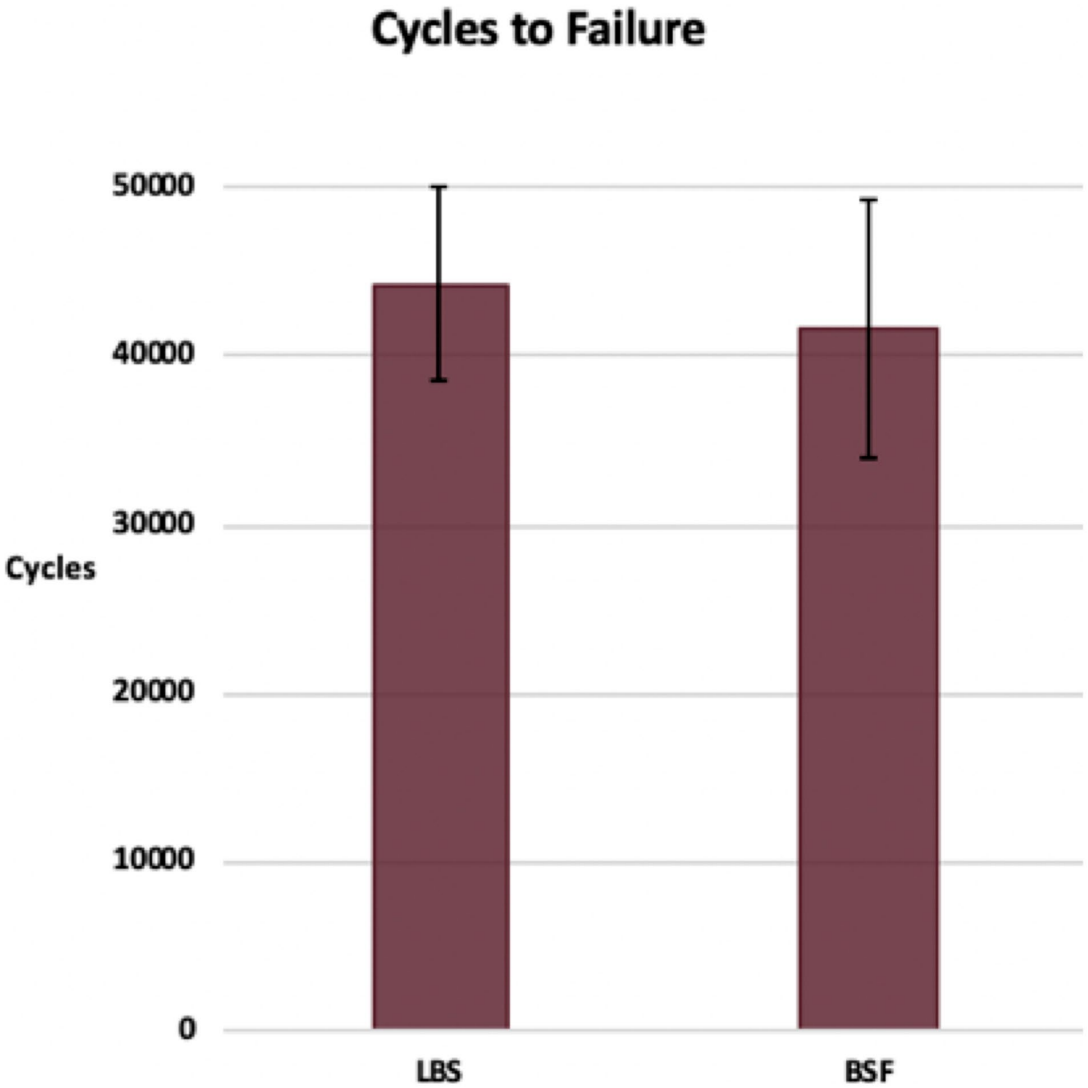
Cycles to failure of the TPLO construct for the locking buttress screw and bone-screw-fastener groups (mean ± SD).

**Figure 4:**
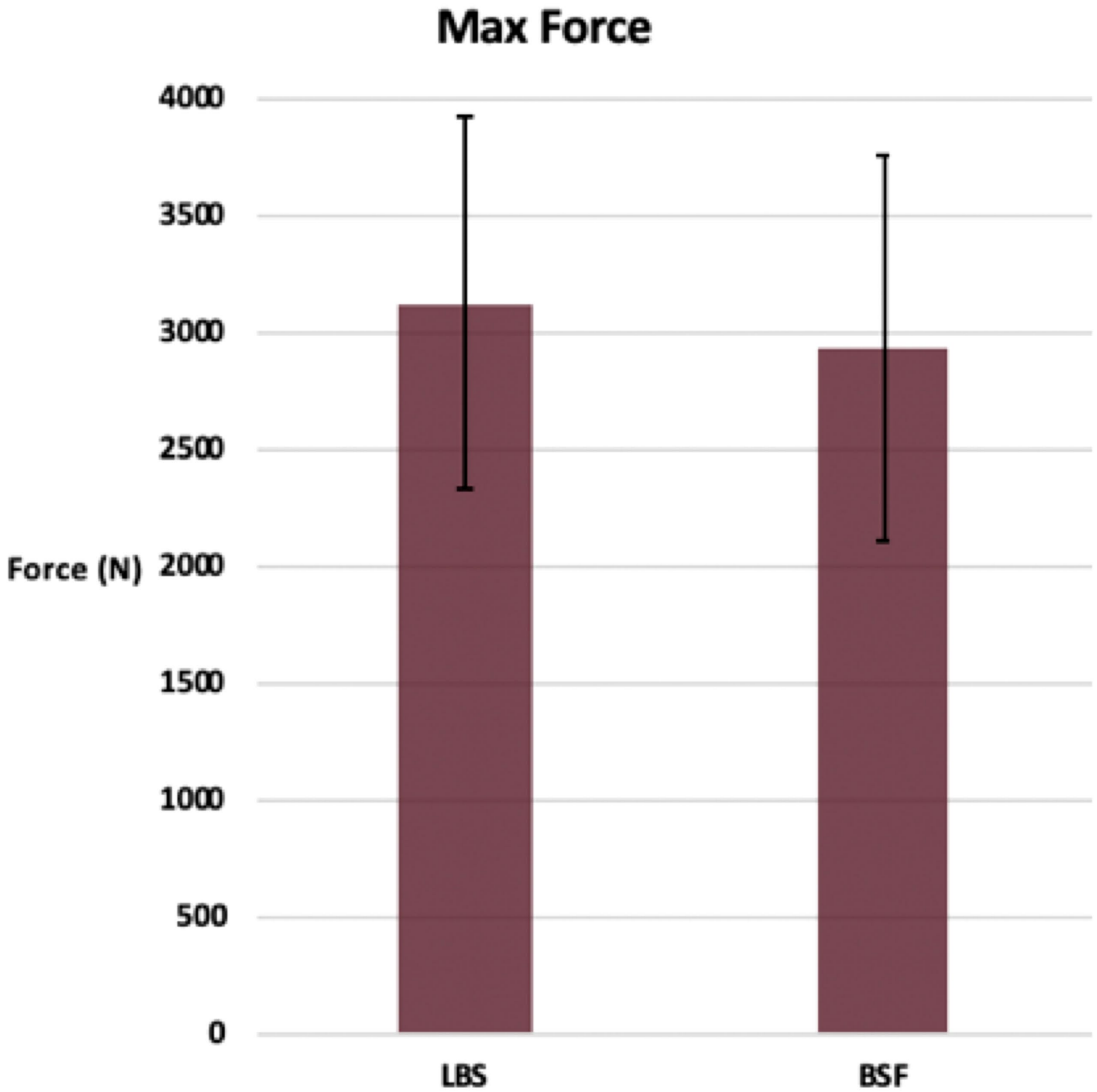
Maximum force prior to failure of the TPLO construct for the locking buttress screw and bone-screw-fastener groups (mean ± SD).

**Figure 5:**
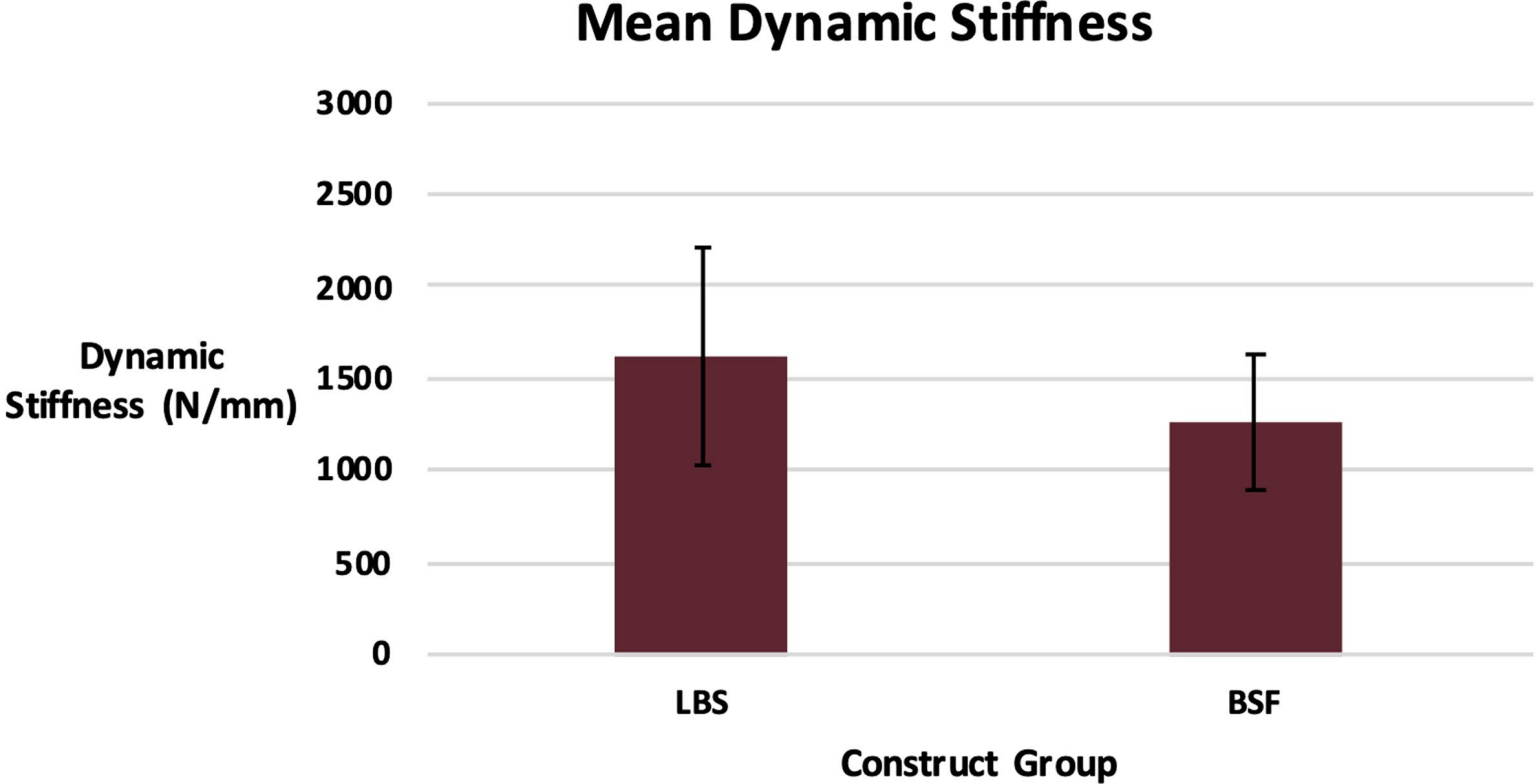
Dynamic stiffness of the TPLO construct for the locking buttress screw and bone-screw-fastener groups for the first 30,000 cycles (mean ± SD).

**Figure 6:**
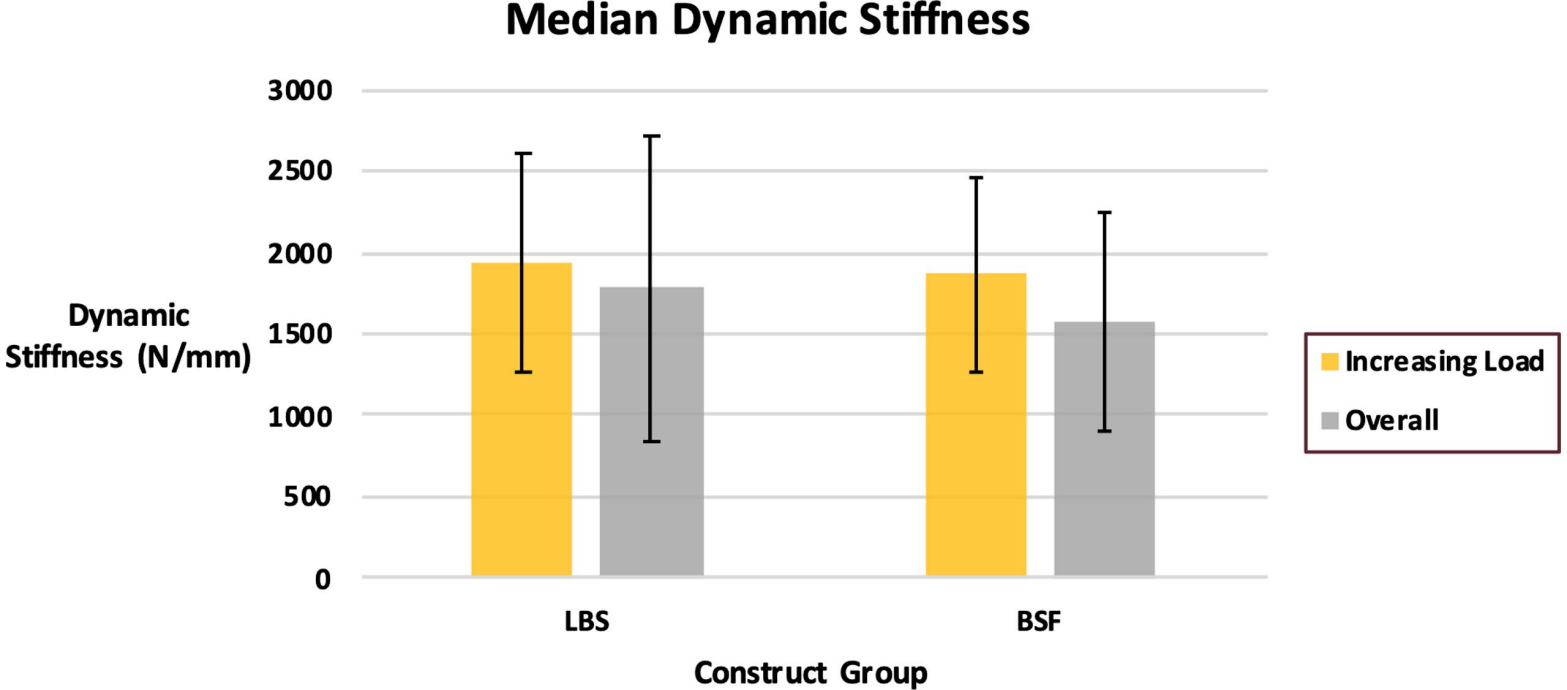
Dynamic stiffness of the TPLO construct for the locking buttress screw and bone-screw-fastener groups for cycles with stepwise increasing load and overall (median ± quartile range).

Cycles to failure for the LBS group (44,260 ± 5,770) and the BSF group (41,540 ± 7,686) were not significantly different (*p* = 0.36) (Figure 3). Maximum forces withstood by the LBS group (3,134 ± 797 N) and the BSF group (2,940 ± 831 N) were not significantly different either (*p* = 0.58) (Figure 4). Dynamic stiffness for the LBS group (30,000: 1,622 ± 586 N/mm; increasing load: 1,936 ± 662 N/mm; and overall: 1,778 ± 932 N/mm) and the BSF group (30,000: 1,261 ± 362 N/mm; increasing load: 1,862 ± 594 N/mm; and overall: 1,574 ± 677 N/mm) was not significantly different (30,000: *p* = 0.11; increasing load: *p* = 0.83; and overall: *p* = 0.58) (Figures 5, 6).

**Figure 7:**
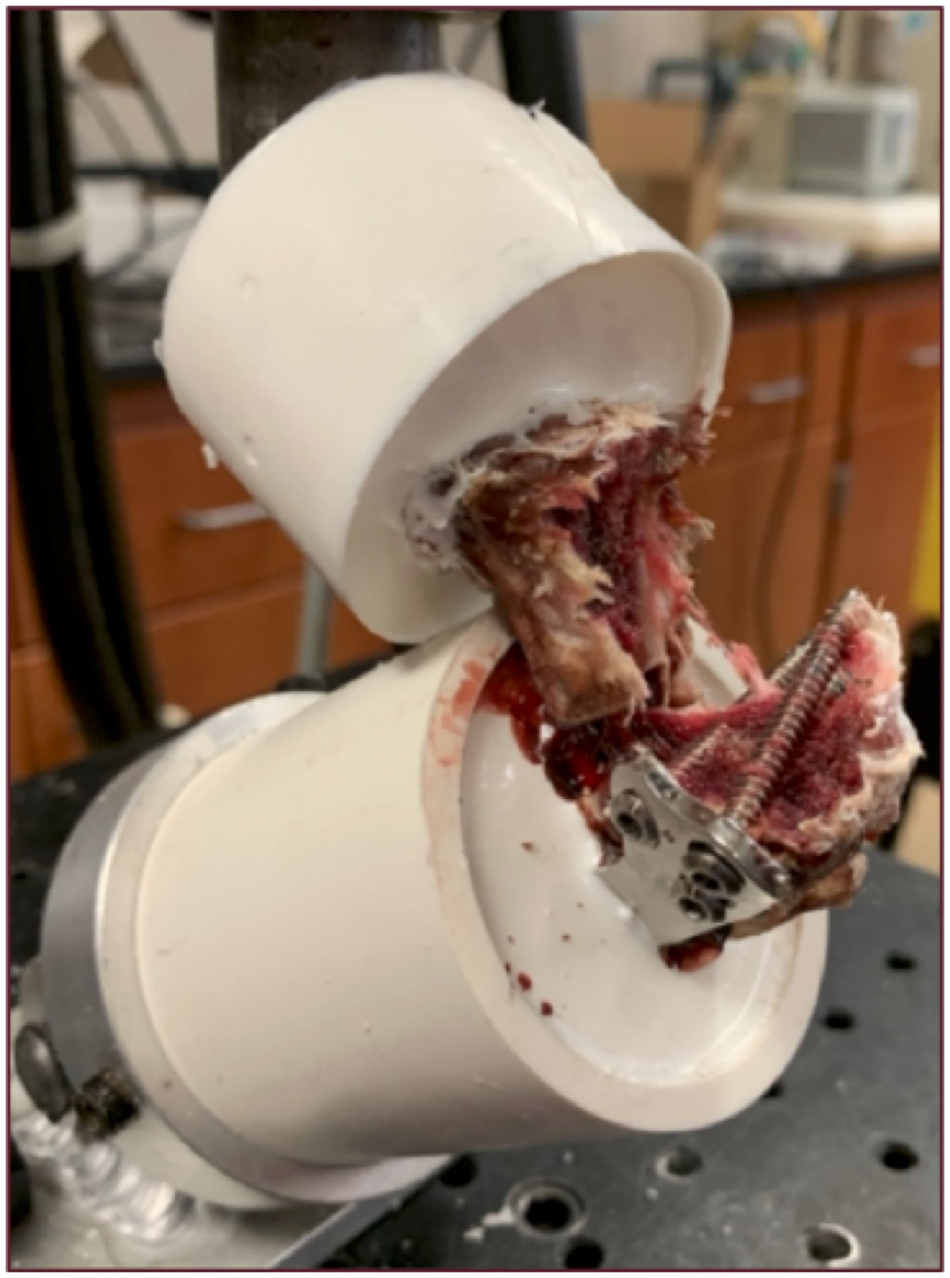
Photograph of fracture of the proximal tibial segment through the screw holes.

Failure was most commonly noted to be associated with loss of rotation of the tibial plateau segment (Table 1). Plastic deformation with no visible evidence of failure was the second most common mode of failure. Fracture of the proximal tibial segment (Figure 7), screw breakage, and screw loosening occurred in both groups. No screws were noted to penetrate the articular surface or osteotomy.

**Table 1:** Modes of failure of the TPLO construct for the locking buttress screw and bone-screw-fastener groups.

Mode of failure	Locking buttress screw	Bone-screw-fastener
Plastic deformation (6 mm)	3	4
Screw breakage	1	0
Screw loosening	1	1
Loss of rotation	5	5
Fracture	2	2

## Discussion

No significant differences were detected between the LBS group and the BSF group regarding cycles to failure, maximum force withstood, or dynamic stiffness of the construct. Therefore, the authors’ hypothesis was rejected. Nevertheless, the findings of this current study suggest that stabilization of the TPLO with bone-screw-fasteners provided similar biomechanical stability under cyclic axial loading conditions as the locking buttress screw.

The setup for mechanical testing was similar to that described by Leitner et al., which aimed to evaluate the long-term effects of dynamic loading on a TPLO construct during the stance phase by axially compressing the construct in a servohydraulic testing machine (8). In that study, constructs were loaded via proximally and distally oriented metal spheres. This setup was intended to simulate *in vivo* loading, ensure joint bending moments of zero, and mimic a worst-case scenario by producing an unstable proximal articular environment. Rotation of the specimen was prevented by insertion of a pin into the distal embedded portion of the construct and guiding it into a vertical slot (8). In this current study, the distal sphere and pin were replaced with a metal rod and sleeve bearings; the effect of these changes was not investigated. A model of the stifle and tarsocrural joints *in situ* and with simulated quadriceps and gastrocnemius muscle groups has been described (7, 17). While models of this nature are utilized to evaluate the effects of the intervention on pelvic limb and joint function, the models used in this study and by Leitner et al. are focused on the relative movements of the bone fragments and tibial osteotomy stability (8).

All constructs were embedded in polyurethane casting resin proximally at the tibial articular surface and distally at the mid diaphysis of the tibia. The plate shaft and distal three screws were embedded to a height of exactly 1 cm distal to the osteotomy. This protocol was selected to solely evaluate the proximal aspect of the TPLO construct. In the study by Leitner et al., 50% of pilot constructs demonstrated splitting of the lateral tibial cortex with propagation along the distal screw holes (8). Because of the focus of this current study, the authors elected to employ an embedding technique that prevented distal failure. In doing so, a worst-case scenario was simulated at the study’s area of interest by concentrating stress at the proximal part of the construct. Despite these limitations, the model used in this study represents an appropriate, albeit restricted, approximation to *in vivo* loading (8). Screw type in the distal three holes differed due to availably of donated implants for this study; the authors believed that this difference would not affect the results of the study, as these screws were embedded in potting material.

Load levels and testing frequency used in this study were significantly higher than loads generated *in vivo* by a walking dog; however, similar loads and gait frequencies may be encountered in large- and giant-breed dogs under extreme conditions (8, 18–22). Thus, this study aimed to test constructs at the highest probable level of stress to determine fatigue points and hence differences between TPLO study groups (8). No significant differences were detected between the LBS and BSF groups under these intense loading conditions.

Five modes of failure were observed in this study: plastic deformation (6 mm crosshead displacement at unloading of 50 N), screw breakage, screw loosening, loss of rotation of the tibial plateau segment, and fracture (Table 1) (8). Failure was most commonly associated with loss of rotation of the tibial plateau segment, indicated by inability to maintain contact between the servohydraulic testing machine, metal sphere, and proximal embedding. Fracture of the proximal tibial fragment occurred through screw holes in 17% (4/24) of constructs (Figure 7). Screw loosening, noted by comparing pre- and post-testing photographs, occurred in 8% (2/24) of constructs. Screw breakage distal to the head of the screw was noted in only a single BSF construct. Plastic deformation with no visible evidence of failure occurred in 29% of constructs (7/24). No difference in frequency of failure modes was detected between groups.

Loss of rotation of the tibial plateau segment occurred in 42% (10/24) of constructs in this study. Changes in TPA during osteotomy healing have been reported following TPLO (8, 23–26). Use of locking screws has been shown to provide superior maintenance of tibial plateau position (8, 23). The LBS group and the BSF group were equally affected by loss of rotation in the current study; however, the magnitude of change in position of the tibial plateau was not quantified. In the study by Leitner et al., titanium pins were utilized to track bone fragment location by collecting computed tomography (CT) images at three separate time points: after reduction of the osteotomy, after plate application, and after 30,000 cycles of axal compression. Doing so demonstrated a significantly greater magnitude of rotation about the sawing axis and translation of the tibial plateau segment toward the bone plate for conventional screws (cortical buttress screws) than for locking screws (8). Use of CT imaging may help evaluate the ability of the bone-screw-fastener to maintain tibial plateau position.

The bone-screw-fastener was designed to resist multidirectional loading, among other theoretical advantages to the conventional buttress screw (9). Use of bone-screw-fasteners may result in superior biomechanical stability following the TPLO procedure under other loading conditions, such as bending or torsion; however, this was beyond the scope of this current study. Further evaluation of the utility of the BSF for the TPLO under these conditions is indicated.

Another theoretical advantage of the bone-screw-fastener or interlocking thread screw is the presence of osteolocking threads that circumferentially interlock with bone (10, 11). While this screw is not a locking screw in the traditional sense (i.e., threaded head), the authors wished to compare the bone-screw-fastener to a locking buttress screw as if both had locking capability. Therefore, the TPLO plates were not contoured to the tibia for either group. Whether or not bone plates should be anatomically contoured when used with bone-screw-fasteners, as is customary with non-locking screws, is unknown. This question warrants additional testing before contouring recommendations can be made for *in vivo* use.

Screw type did not affect TPLO stability under cyclic loading conditions in this current study. However, it is possible given our sample size that a significant difference may have not been detectable due to our study’s power. While a pre-hoc power analysis was not performed, this study included 50% more samples than the study by Leitner et al. (8).

Limitations of this study include use of an *ex vivo* model and limited sample size. As mentioned previously, testing was restricted to axial compression. In addition, constructs only included large-breed dogs and 3.5 mm implants; whether results would differ in toy- or small-breed dogs or with smaller implants is unknown. Future research endeavors should focus on testing the bone-screw-fastener under other loading conditions and exploring stabilization of smaller TPLO plates with these screws.

The findings of this current study suggest that stabilization of the TPLO with bone-screw-fasteners provided no advantage to biomechanical stability under cyclic axial loading conditions when compared to the locking buttress screw. Bone-screw-fasteners may be an acceptable alternative to traditional locking screws for TPLO. Further research, particularly *in vivo* experiments, is warranted prior to clinical application.

## Data availability statement

The raw data supporting the conclusions of this article will be made available by the authors, without undue reservation.

## Author contributions

WK, MJ, RW, SD, and SE contributed to the conception of study, study design, acquisition of data, and data analysis and interpretation. Figure 1 was illustrated by WK. All authors contributed to the article and approved the submitted version.

## Funding

This study was funded by the Mississippi State University College of Veterinary Medicine Office of Research and Graduate Studies through the House Officer Clinical Research Grant Program.

## Conflict of interest

The authors declare that the research was conducted in the absence of any commercial or financial relationships that could be construed as a potential conflict of interest.

## Publisher’s note

All claims expressed in this article are solely those of the authors and do not necessarily represent those of their affiliated organizations, or those of the publisher, the editors and the reviewers. Any product that may be evaluated in this article, or claim that may be made by its manufacturer, is not guaranteed or endorsed by the publisher.
